# Trivalent Polyhedra as Duals of Borane Deltahedra: From Molecular Endohedral Germanium Clusters to the Smallest Fullerenes

**DOI:** 10.3390/molecules28020496

**Published:** 2023-01-04

**Authors:** R. Bruce King

**Affiliations:** Department of Chemistry, University of Georgia, Athens, GA 30602, USA; rbking@uga.edu

**Keywords:** dualization of polyhedra, borane deltahedra, endohedral germanium clusters, small fullerenes

## Abstract

The duals of the most spherical *closo* borane deltahedra having from 6 to 16 vertices form a series of homologous spherical trivalent polyhedra with even numbers of vertices from 8 to 28. This series of homologous polyhedra is found in endohedral clusters of the group 14 atoms such as the endohedral germanium cluster anions [M@Ge_10_]^3−^ (M = Co, Fe) and [Ru@Ge_12_]^3−^ The next members of this series have been predicted to be the lowest energy structures of the endohedral silicon clusters Cr@Si_14_ and M@Si_16_ (M = Zr, Hf). The largest members of this series correspond to the smallest fullerene polyhedra found in the endohedral fullerenes M@C_28_ (M = Zr, Hf, Th, U). The duals of the oblate (flattened) ellipsoidal deltahedra found in the dirhenaboranes Cp*_2_Re_2_B*_n_*_−2_H*_n_*_−2_ (Cp* = η^5^-Me_5_C_5_; 8 ≤ *n* ≤ 12) are prolate (elongated) trivalent polyhedra as exemplified experimentally by the germanium cluster [Co_2_@Ge_16_]^4−^ containing an endohedral Co_2_ unit.

## 1. Introduction

The fundamental structural units for polyhedral boranes, including the B*_n_*H*_n_*^2−^ dianions as well as the isoelectronic carboranes CB*_n_*_−1_H*_n_*^−^ and C_2_B*_n_*_−2_H*_n_*, are the most spherical *closo* deltahedra in which all of the faces are triangles (Table 1). [1,2,3] Such deltahedra are also the structural units for many other types of cluster compounds, including transition metal and post-transition element clusters. Thus bare group 14 element vertices (Si, Ge, Sn, Pb) as well as Fe(CO)_3_ and (η^5^-C_5_H_5_)Co vertices are valence isoelectronic and isolobal with BH vertices. Similarly bare group 15 element vertices (P, As, Sb, Bi) vertices as well as Co(CO)_3_ and (η^5^-C_5_H_5_)Ni vertices are valence isoelectronic and isolobal with CH vertices. The duals of these most spherical deltahedra represent a series of most spherical trivalent polyhedra in which the degree 3 vertices correspond to the triangular faces of the most spherical deltahedra. This paper surveys the wide range of cluster structures ranging from molecular endohedral germanium clusters to the smallest endohedral fullerenes based on trivalent polyhedra that are duals of the deltahedra found in polyhedral borane and metallaborane chemistry.

The most spherical *closo* deltahedra have the following features:

(1) All faces are triangles—hence their designation as deltahedra relating to the shape of the Greek letter delta (∆). This feature maximizes the number of edges for a given number of vertices and thus maximizes the connectivity between the vertices.
(2) The vertices are as nearly similar as possible thereby providing the best approximation to a sphere rather than a prolate or oblate ellipsoid.

**Figure 1:**
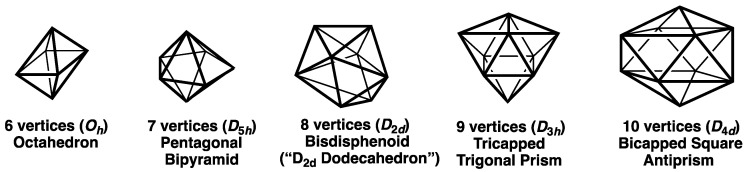
The most spherical *closo* polyhedra having 6 to 10 vertices.

**Figure 2:**
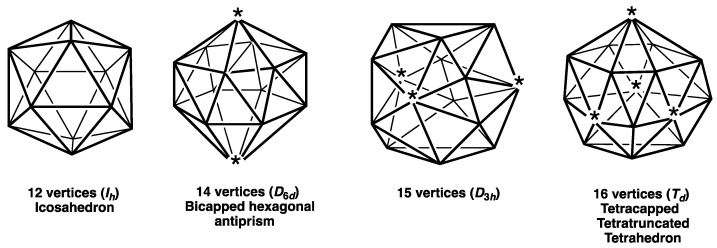
The four Frank-Kasper deltahedra with the degree 6 vertices starred.

**Figure 3:**
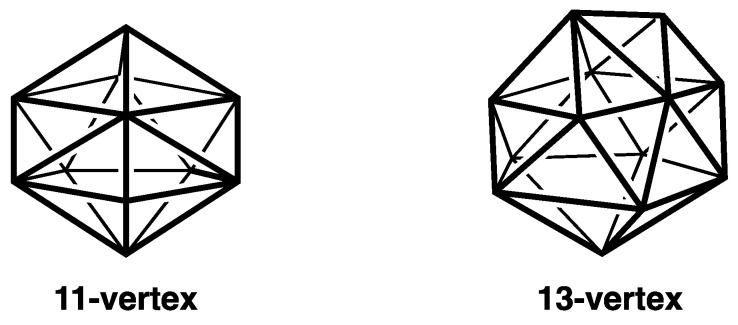
The 11- and 13-vertex *closo* deltahedra.

With these features in mind, the most spherical *closo* deltahedra can be classified into the following three types:

(3) Deltahedra with exclusively degree 4 and 5 vertices where the degree of a given vertex is the number of edges meeting at that vertex (Figure 1). These start with the highly symmetrical (*O_h_*) 6-vertex regular octahedron with exclusively degree 4 vertices and go as far as the *D*_4*d*_ 10-vertex bicapped square antiprism with two degree 4 vertices and eight degree 5 vertices. These deltahedra are found in the borane dianions B*_n_*H*_n_*^2−^ and the isoelectronic carboranes CB*_n_*_−1_H*_n_*^−^ and C_2_B*_n_*_−2_H*_n_* [1,2,3].
(4) The four Frank-Kasper deltahedra [4] with exclusively degree 5 and degree 6 vertices with no pair of degree 6 vertices sharing an edge (Figure 2). These start with the highly symmetrical (*I_h_*) 12-vertex regular icosahedron and also include the 14-vertex *D*_6*d*_ bicapped hexagonal antiprism with antipodal degree 6 vertices, a 15-vertex *D*_3*h*_ deltahedron with its three degree 6 vertices forming an equilateral macrotriangle, and the 16-vertex *T_d_* tetracapped tetratruncated tetrahedron with its four degree 6 vertices forming a regular macrotetrahedron. The icosahedral structure is found in the borane dianion B_12_H_12_^2−^ as well as the isoelectronic carboranes CB_11_H_12_^−^ and C_2_B_10_H_12_ [1,2,3]. The 14-vertex bicapped hexagonal antiprism is found in (µ-CH_2_)_3_C_2_B_12_H_12_ in which the two carbon vertices are bridged by a trimethylene unit [5]. The 16-vertex tetracapped tetratruncated tetrahedron is found in the unusual pentarhodium complex (Cp*Rh)_3_B_12_H_12_Rh(B_4_H_9_ RhCp*) (Cp* = η^5^-Me_5_C_5_) [6].
(5) For the 11- and 13-vertex systems the *closo* deltahedra are less symmetrical and necessarily contain vertices of three different degrees, namely 4, 5, and 6 (Figure 3).

The 16-vertex Frank-Kasper deltahedron is a natural upper limit to the number of vertices in this homologous series of most spherical deltahedra. Thus deltahedra having more than 16 vertices require adjacent degree 6 vertices and/or vertices of degrees higher than 6. Adjacent degree 6 vertices lead to “flat spots” on the surface of the deltahedron and vertices of degrees higher than 6 can even lead to indentations (“negative curvature”) on the polyhedral surface. Either of these features leads to significant distortion from sphericality.

The initially synthesized group 14 element clusters containing interstitial atoms have deltahedral structures for the outer group 14 shell. Thus in the trianions [Cu@E_9_]^3−^ (E = Sn, Pb) a copper atom is encapsulated by a *closo* 9-vertex tricapped trigonal prismatic tin or lead cluster [7]. Similarly, the *closo* 10-vertex *D*_4*h*_ bicapped square antiprism encapsulating a metal atom was found to be the structural motif for the anionic indium cluster Zn@In_10_^8−^ found in the intermetallic [8] K_8_In_10_Zn as well as in the lead clusters M@Pb_10_^2−^ found in [K(2,2,2-crypt)]_2_[M@Pb_10_] (M = Ni, Pd, Pt). [9,10] The other 10-vertex polyhedra, such as the *C*_3*v*_ tetracapped trigonal prism in the M@Ga_10_^10−^ clusters found in the K_10_Ga_10_M intermetallics (M = Ni, Pd, Pt) [11] and the pentagonal antiprism in the cationic bismuth cluster Pd@Bi_10_^4+^ in Bi_14_PdBr_16_ (=[Pd@Bi_10_][BiBr_4_]_4_), [12] have all or at least mostly triangular faces. The dianion [Pt@Pb_12_]^2−^ contains a platinum atom encapsulated in a *closo* regular icosahedron of lead atoms. [13]

In view of these considerations the discovery of an outer Ge_10_ pentagonal prism in the anion Co@Ge_10_^3−^ of [K(2,2,2-crypt)]_4_[Co@Ge_10_][Co(1,5-C_8_H_12_)_2_]•toluene [14] and in the anion Fe@Ge_10_^3−^ of [K(2,2,2-crypt)]_3_[Fe@Ge_10_] [15] was a major surprise. Not only is the pentagonal prism not a most spherical deltahedron or even any kind of deltahedron, it does not have any triangular faces at all. I now show how the pentagonal prism, as the dual of the pentagonal bipyramid, is the second member of a homologous series of trivalent polyhedra starting with the cube and ending with the *T_d_* 28-vertex polyhedron found in the endohedral fullerenes M@C_28_ (M = Zr, Th, U). [16] In this connection, a trivalent polyhedron is defined as a polyhedron in which all vertices have degree 3. Such trivalent polyhedra must necessarily have an even number of vertices as well as the minimum number of edges for a polyhedron with a given number of vertices. Minimizing the number of edges for a given number of vertices maximizes the internal volume of the polyhedron by minimizing the connectivity between the vertices. Therefore, trivalent polyhedra are especially suitable as maximum-volume containers for interstitial atoms.

**Figure 4:**
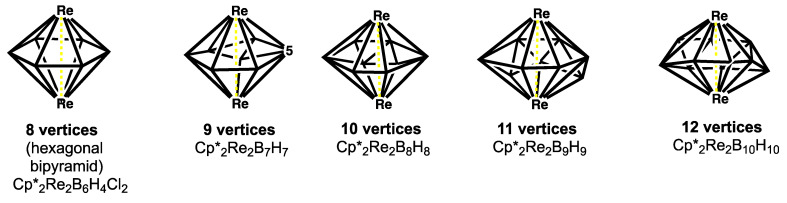
The oblate ellipsoidal (*oblatocloso*) deltahedra found in the dirhenaboranes Cp*_2_Re_2_B*_n_*_−2_H*_n_*_−2_ (Cp* = η^5^-Me_5_C_5_; 8 ≤ *n* ≤ 12).

In addition to the most spherical borane deltahedra (Table 1 and Figure 1) oblate (flattened) ellipsoidal deltahedra are found in the so-called *oblatocloso* dirhenaboranes Cp*_2_Re_2_B*_n_*_−2_H*_n_*_−2_ (Cp* = η^5^-Me_5_C_5_; 8 ≤ *n* ≤ 12) (Figure 4). [17,18,19,20] The flattened shape of these deltahedra arises from the need to bring the two rhenium atoms in approximately antipodal positions close enough to form a rhenium-rhenium bond through the center of the deltahedron. [21] The duals of such oblate deltahedra are prolate elongated trivalent polyhedra such as the 16-vertex polyhedron found experimentally in the germanium cluster [Co_2_@Ge_16_]^4−^ with an endohedral Co_2_ unit. [22,23]

## 2. Dualization of Polyhedra

**Figure 5:**
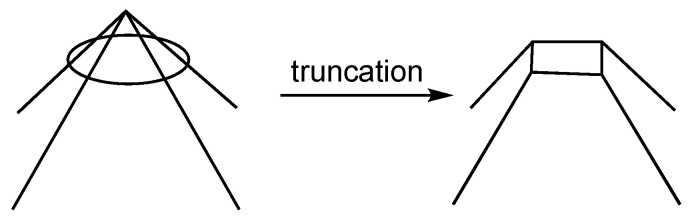
Schematic representation of the truncation of a degree 4 vertex to give a new tetragonal face.

The dual of a polyhedron has its vertices located at the midpoints of the faces of the original polyhedron. Two vertices of the dual are connected by an edge if the corresponding faces of the original polyhedron share an edge. The dualization of a polyhedron preserves the symmetry of the original polyhedron. Furthermore, the dualization of the dual of a polyhedron leads back to the original polyhedron. In this sense, the process of dualization has a period of two so that polyhedra occur in natural pairs of a given polyhedron and its dual. Among the five regular polyhedra the octahedron and cube form a dual pair exhibiting *O_h_* symmetry and the icosahedron and dodecahedron form a dual pair exhibiting *I_h_* symmetry. The tetrahedron is a self-dual polyhedron since the dual of a tetrahedron is another tetrahedron. Self-dual polyhedra necessarily have the same number of vertices and faces. In this connection, pyramids are self-dual polyhedra. The dual of a bipyramid is a prism of the same symmetry and vice versa. The dual of an antiprism is a trapezohedron of the same symmetry and vice versa,

A number of chemically significant polyhedra are derived by capping one or more faces of smaller polyhedra. Examples among the most spherical *closo* deltahedra include the 9-vertex *D*_3*h*_ tricapped trigonal prism and the 10-vertex *D*_4*d*_ bicapped square antiprism (Figure 1). The dual process of capping is truncation (Figure 5). Truncation consists of cutting off a vertex to generate a new polygonal face with a number of sides corresponding to the degree of the cut-off vertex. Thus the dual of the *D*_3*h*_ tricapped trigonal prism is the 14-vertex tritruncated trigonal bipyramid, also of *D*_3*h*_ symmetry. Similarly, the dual of the *D*_4*d*_ bicapped square antiprism is the bitruncated square trapezohedron, also of *D*_4*d*_ symmetry. Dualization of the 16-vertex tetracapped tetratruncated tetrahedron, namely the 16-vertex Frank Kasper deltahedron (Figure 2), gives the tetratruncated tetracapped tetrahedron. In this case, dualization consists of reversing the order of tetracapping and tetratruncating the original tetrahedron.

## 3. The Most Spherical Trivalent Polyhedra in Chemistry

**Figure 6:**
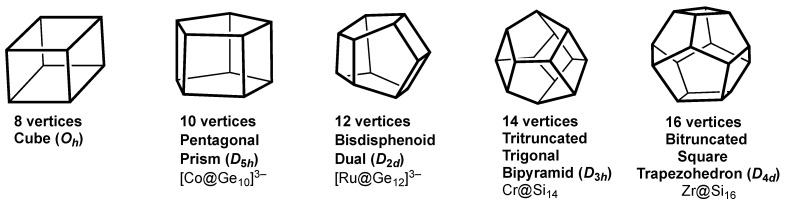
The most spherical trivalent polyhedra with 8, 10, 12, 14, and 16 vertices as duals of the *closo* deltahedra in endohedral Group 14 clusters.

**Figure 7:**
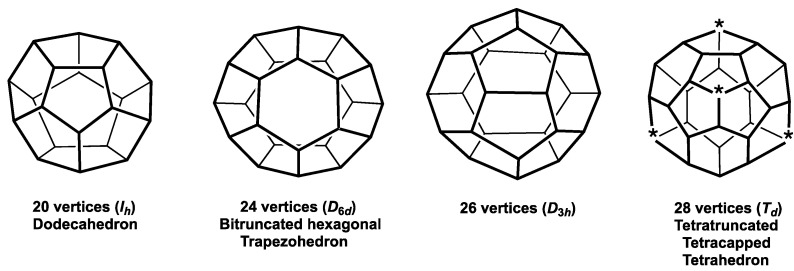
The most spherical trivalent polyhedra having 20, 24, 26, and 28 vertices as duals of the Frank-Kasper deltahedra (Figure 2). In the *T_d_* 28-vertex polyhedron the four degree 3 vertices each shared by three pentagonal faces are starred to indicate their orientation in M@C_28_ species.

The duals of the most spherical *closo* deltahedra form a natural series of most spherical maximum volume trivalent polyhedra starting with the 8-vertex cube and ending with the 28-vertex tetratruncated tetracapped tetrahedron (Table 1). The duals of the 6- to 10-vertex *closo* deltahedra (Figure 1) have exclusively tetragonal and pentagonal faces (Figure 6). Similarly, the duals of the four Frank-Kasper deltahedra (Figure 2) have exclusively pentagonal and hexagonal faces (Figure 7). The duals of the less symmetrical and thus less distinctive 11- and 13-vertex *closo* deltahedra (Figure 3) necessarily have three different types of faces, namely tetragonal, pentagonal, and hexagonal faces. They have not been identified in any chemical structures and thus are not discussed in this paper.

The group 14 elements (C, Si, Ge, Sn, Pb) are well suited to be vertices of trivalent polyhedra. The four tetrahedrally disposed sp^3^ orbitals of each group 14 element vertex allow for three two-center two-electron bonds along each of the polyhedral edges connected to that vertex leaving an external sp^3^ hybrid for a lone pair. The maximum volume feature of the most spherical trivalent polyhedra is important in order to have a large enough cavity to accommodate an interstitial atom. The smallest members of the trivalent polyhedral homologous series allow germanium clusters to accommodate an interstitial transition metal atom. This is where the pentagonal prismatic [M@Ge_10_]^3−^ (M = Co, [14] Fe [15]) trianions fit into the picture. Similarly the 12-vertex bisdisphenoid dual is found in the [Ru@Ge_12_]^3−^ trianion found in [K(2,2,2-crypt)]_3_[Ru@Ge_12_]·4py [24,25] and in the anion [Ta@Ge_8_As_4_]^3−^, also found in a [K(2,2,2-crypt)]^+^ salt of more complicated stoichiometry [26]. The larger 14-vertex tritruncated trigonal bipyramid is found in the lanthanide-centered [Ln@Sn_7_Bi_7_]^4−^ (Ln = La, Ce) tetraanions [27] and is predicted to be a favorable polyhedron for M@Si_14_ clusters encapsulating first row transition metals, some of which are observed in supersonic beams [28]. The 16-vertex bitruncated square trapezohedron is also found as a silicon cluster encapsulating a heavier group 4 metal atom in low energy M@Si_16_ (M = Zr, Hf) structures [29,30,31].

The most spherical trivalent polyhedra with 20 or more vertices correspond to the smallest C*_n_* fullerenes having only pentagonal and hexagonal faces. Even with carbon vertices, such polyhedra become large enough to encapsulate metal atoms. Thus the 28-vertex tetratruncated tetracapped tetrahedron (Figure 6) can accommodate group 4 metals and actinides in the M@C_28_ species (M = Zr, Th, U) [16]. In these structures the tetrahedral orientation of the four vertices common to three fused pentagonal rings (starred vertices in Figure 6) provide tetrahedral coordination for the encapsulated metal atom.

The M@C_28_ (M = Zr, Th, U) species are the smallest fullerene derivatives that have been realized experimentally. However, the endohedral fullerenes U@C_26_ exhibiting the *D*_3*h*_ most spherical trivalent polyhedral geometry [32] and Pu@C_24_ exhibiting *D*_6*d*_ bitruncated hexagonal trapezohedral geometry [33] (Figure 7) are predicted to be favorable species with high HUMO-LUMO gaps and positive binding energies. The endohedral silicon cluster anion [U@Si_20_]^6−^ with an outer Si_20_ regular (*I_h_*) dodecahedron (Figure 6) is also predicted to be a favorable species by similar criteria [34]. All of these favorable endohedral clusters with geometries based on duals of the Frank-Kasper polyhedra (Figure 6) have 32 skeletal electrons (= 2*n*^2^ for *n* = 4) counting one skeletal electron for each group 14 element vertex and all of the valence electrons of the endohedral atom. This relates to the spherical aromaticity [35] of such systems consisting of filled 1S + 1P + 1D + 1F molecular orbitals leaving a significant HOMO-LUMO gap.

**Table 1:** The most spherical *closo* deltahedra and their duals.

Vertices/		*Faces/*	*Closo Deltahedron*	Dual Trivalent	Chemical Example
Faces (Dual)	Edges	Vertices (Dual)	(Symmetry)	Polyhedron	of Dual ^a^ [lit. ref.]
6	12	8	Octahedron (*O_h_*)	Cube	
7	15	10	Pentag bipyramid (*D*_5*h*_)	Pentag prism	[Co@Ge_10_]^3−^ [14]
8	18	12	Bisdisphenoid (*D*_2*d*_)		[Ru@Ge_12_]^3−^ [15]
9	21	14	Tricap trig prism (*D*_3*h*_)	Tritrunc trig bipyramid	Cr@Si_14_ [28]
10	24	16	Bicap sq antiprism (*D*_4*d*_)	Bitrunc sq trapezohedron	Zr@Si_16_ [29,30,31]
11	27	18	(*C*_2*v*_)		
12	30	20	Icosahedron (*I_h_*)	Dodecahedron	{[U@Si_20_]^6−^} [34]
13	33	22			
14	36	24	Bicap hex antiprism (*D*_6*d*_)	Bitrunc hex trapezohedron	{Pu@C_24_} [33]
15	39	26	15v Frank Kasper (*D*_3*h*_)		{U@C_26_} [32]
16	42	28	Tetracap tetratrunc tet (*T_d_*)	Tetratrunc tetracap tet	Th@C_28_ [16]

^a^ Species predicted theoretically but not yet realized experimentally are listed in braces {}. Literature references are indicated in brackets [].

## 4. Prolate Elongated Ellipsoidal Trivalent Polyhedra as Duals of Oblate Flattened Ellipsoidal Deltahedra

The series of hypoelectronic dirhenaboranes Cp*_2_Re_2_B*_n_*_−2_H*_n_*_−2_ (Cp* = η^5^-Me_5_C_5_; 8 ≤ *n* ≤ 12) have central oblate (flattened) ellipsoidal Re_2_B*_n_*_−2_ deltahedra (Figure 4) that are very different from the most spherical *closo* borane deltahedra (Figure 1) [17,18,19,20]. The flattening arises from the need to bring the two approximately antipodal rhenium atoms close enough (2.69 to 2.94 Å) for a direct Re=Re interaction through the center of the deltahedron [21].

**Figure 8:**
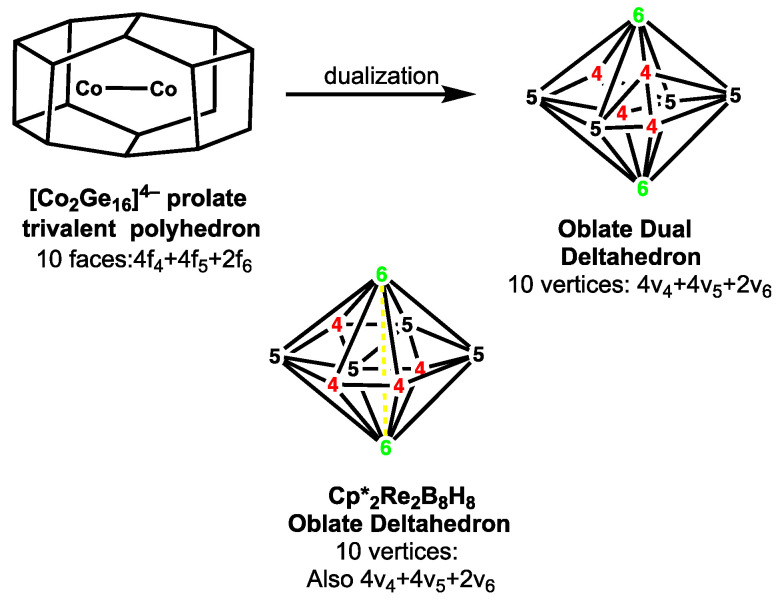
Dual relationship between the 16-vertex prolate ellipsoidal trivalent polyhedron in [Co_2_Ge_16_]^4−^ and the 10-vertex oblate ellipsoidal deltahedron in Cp*_2_Re_2_B_8_H_8_.

Dualization of an oblate (flattened) ellipsoidal deltahedron such as those found in the hypoelectronic dirhenaboranes leads to a prolate (elongated) trivalent polyhedron. The elongated shapes and relatively large internal volumes of such prolate trivalent polyhedra make them particularly suitable to incorporate a pair of interstitial metal atoms. This is seen experimentally in the [Co_2_@Ge_16_]^4−^ tetraanion found in the salt [K(2,2,2-crypt)]_4_[Co_2_@Ge_16_]·en where one of the two isolated and crystallographically characterized isomers has a structure based on a prolate trivalent polyhedron with two hexagonal faces, four pentagonal faces, and four tetragonal faces (Figure 8) [22,23]. The Co–Co distance of 2.75 Å in the endohedral Co_2_ unit of this structure can correspond to a long single bond. This prolate trivalent polyhedron bears an approximate dual relationship with the oblate deltahedron found in the dirhenaborane Cp*_2_Re_2_B_8_H_8_ with two vertices of degree 6, four vertices of degree 5, and two vertices of degree 6. In this case, the duality relationship is not exact since there is a shift of the relative positions of the four degree 5 and four degree vertices in the Cp*_2_Re_2_B_8_H_8_ structure in order to place the pair of rhenium atoms at the optimum distance for the intrapolyhedral rhenium-rhenium bonding.

## 5. Conclusions

The duals of the most spherical *closo* borane deltahedra having from 6 to 16 vertices form a series of homologous spherical trivalent polyhedra having even numbers of vertices from 8 to 28. This series of homologous polyhedra is found in endohedral clusters of the group 14 atoms. Thus the smallest members of this series are found in the endohedral germanium cluster anions [M@Ge_10_]^3−^ (M = Co, Fe) and [Ru@Ge_12_]^3−^ with *D*_5*h*_ pentagonal prism and *D*_2*d*_ bisdisphenoid dual geometries, respectively. The next members of this series have been predicted to be the lowest energy structures of the endohedral silicon clusters Cr@Si_14_ and M@Si_16_ (M = Zr, Hf) with *D*_3*h*_ tritruncated trigonal bipyramid and *D*_4*d*_ bitruncated tetragonal trapezohedron geometries, respectively. The largest members of this series correspond to the smallest fullerene polyhedra. The last member of this series, namely the *T_d_* tetratruncated tetracapped tetrahedron dual to the 16-vertex Frank-Kasper deltahedron, is found in the endohedral fullerenes M@C_28_ (M = Zr, Hf, Th, U), which are the smallest fullerene polyhedra known experimentally. Thus the homologous series of most spherical trivalent polyhedra represent a transition from molecular endohedral clusters of the heavier group 14 elements to the smallest fullerene derivatives. In addition, the duals of the oblate (flattened) ellipsoidal deltahedra found in the dirhenaboranes Cp*_2_Re_2_B*_n_*_−2_H*_n_*_−2_ (Cp* = η^5^-Me_5_C_5_; 8 ≤ *n* ≤ 12) are prolate (elongated) trivalent polyhedra as exemplified experimentally by the germanium cluster [Co_2_@Ge_16_]^4−^ with an endohedral Co_2_ unit.

## Data Availability

Not applicable.
